# Orthosteric Binding of ρ-Da1a, a Natural Peptide of Snake Venom Interacting Selectively with the α_1A_-Adrenoceptor

**DOI:** 10.1371/journal.pone.0068841

**Published:** 2013-07-25

**Authors:** Arhamatoulaye Maïga, Jon Merlin, Elodie Marcon, Céline Rouget, Maud Larregola, Bernard Gilquin, Carole Fruchart-Gaillard, Evelyne Lajeunesse, Charles Marchetti, Alain Lorphelin, Laurent Bellanger, Roger J. Summers, Dana S. Hutchinson, Bronwyn A. Evans, Denis Servent, Nicolas Gilles

**Affiliations:** 1 Commissariat à l'énergie atomique et aux énergies alternatives, iBiTec-S, Service d'Ingénierie Moléculaire des Protéines, Gif sur Yvette, France; 2 Department of Pharmacology, Monash Institute of Pharmaceutical Sciences, Monash University, Parkville, Victoria, Australia; 3 Drug Discovery Biology, Monash Institute of Pharmaceutical Sciences, Monash University, Parkville, Victoria, Australia; 4 Commissariat à l'énergie atomique et aux énergies alternatives, iBiTec-S, Service de Bioénergétique, Biologie Structurale et Mécanismes, Gif sur Yvette, France; 5 Commissariat à l'énergie atomique et aux énergies alternatives, iBEB, Service de Biochimie et Toxicologie Nucléaire, Bagnols-sur-Cèze Cedex, France; University of South Florida College of Medicine, United States of America

## Abstract

ρ-Da1a is a three-finger fold toxin from green mamba venom that is highly selective for the α_1A_-adrenoceptor. This toxin has atypical pharmacological properties, including incomplete inhibition of ^3^H-prazosin or ^125^I-HEAT binding and insurmountable antagonist action. We aimed to clarify its mode of action at the α_1A_-adrenoceptor. The affinity (pKi 9.26) and selectivity of ρ-Da1a for the α_1A_-adrenoceptor were confirmed by comparing binding to human adrenoceptors expressed in eukaryotic cells. Equilibrium and kinetic binding experiments were used to demonstrate that ρ-Da1a, prazosin and HEAT compete at the α_1A_-adrenoceptor. ρ-Da1a did not affect the dissociation kinetics of ^3^H-prazosin or ^125^I-HEAT, and the IC_50_ of ρ-Da1a, determined by competition experiments, increased linearly with the concentration of radioligands used, while the residual binding by ρ-Da1a remained stable. The effect of ρ-Da1a on agonist-stimulated Ca^2+^ release was insurmountable in the presence of phenethylamine- or imidazoline-type agonists. Ten mutations in the orthosteric binding pocket of the α_1A_-adrenoceptor were evaluated for alterations in ρ-Da1a affinity. The D106^3.32^A and the S188^5.42^A/S192^5.46^A receptor mutations reduced toxin affinity moderately (6 and 7.6 times, respectively), while the F86^2.64^A, F288^6.51^A and F312^7.39^A mutations diminished it dramatically by 18- to 93-fold. In addition, residue F86^2.64^ was identified as a key interaction point for ^125^I-HEAT, as the variant F86^2.64^A induced a 23-fold reduction in HEAT affinity. Unlike the M1 muscarinic acetylcholine receptor toxin MT7, ρ-Da1a interacts with the human α_1A_-adrenoceptor orthosteric pocket and shares receptor interaction points with antagonist (F86^2.64^, F288^6.51^ and F312^7.39^) and agonist (F288^6.51^ and F312^7.39^) ligands. Its selectivity for the α_1A_-adrenoceptor may result, at least partly, from its interaction with the residue F86^2.64^, which appears to be important also for HEAT binding.

## Introduction

Many toxins that interact with voltage- and ligand-gated ion channels display both high affinity and selectivity. For the last 50 years, these properties have been used to identify, purify and classify membrane targets and for structure/function studies. The particular properties of these toxins are now also being exploited pharmacologically, and some toxins are used as drugs and others are currently undergoing preclinical trials [1]–[4].

Although voltage- and ligand-gated ion channels are the main targets for neurotoxins, other targets, including G Protein-Coupled Receptors (GPCRs), have also been identified. The animal toxins active on GPCRs can be divided into two families [5]. Members of the first family, the sarafotoxins, conopressin or contulakin-G mimic the natural agonist of the targeted receptor: endothelin, vasopressin and neurotensin, respectively. The second family consists of highly reticulated toxins with folds that are unrelated to any natural ligands. Nine have been isolated from mamba venoms and are active against muscarinic acetylcholine receptors and adrenoceptors (ARs), [6]. Two other toxins: ρ-TIA, from *Conus tulipa* and β-cardiotoxin, from the snake *Ophiophagus hannah*, are active against α_1_-ARs [7] and β-ARs [8], respectively. We suspected that animal venoms are a potential source of novel GPCR binding agents, and developed a screening strategy, initially focused on the binding of green mamba venom to ARs. This screening led to the isolation of two novel snake toxins from *Dendroaspis angusticeps*: ρ-Da1a, previously called AdTx1, which is highly selective for the α_1A_-AR [9], and ρ-Da1b, selective for α_2_-ARs [10]. ρ-Da1a and ρ-Da1b are peptides of 65 and 66 residues, respectively, reticulated by four disulfide bridges, and are members of the three-finger-fold toxin family. The modes of action of these peptide ligands on ARs are not clear. In equilibrium binding experiments, neither ρ-Da1a nor ρ-Da1b fully inhibits radioligand binding [9],[10]. In addition, in isolated prostatic muscle, ρ-Da1a acts as an insurmountable antagonist [9], and cell-based assays indicate that ρ-Da1b is a non-competitive antagonist at the human α_2A_-AR [10].

Adrenergic and muscarinic toxins isolated from mamba snake venoms belong to the same three-finger-fold family and display substantial sequence identity (52–97%) [5]. The interactions between MT1, MT7 and M1 muscarinic receptors have been studied in detail [11]–[14]. Pharmacological studies indicate competition between MT1 and ^3^H-N- methylscopolamine [11], [14], [15]. In contrast, MT7 significantly affects the dissociation kinetics of ^3^H-N- methylscopolamine and ^3^H-acetylcholine [14], [16] and leaves residual binding in equilibrium binding experiments [11] suggesting an allosteric mode of action. As a negative allosteric modulator, MT7 reduces the efficacy and potency of carbamylcholine at M1 muscarinic receptors expressed in CHO cells [16] and interacts mainly with the extracellular loop 2 of this receptor [13], [17]. The smallest peptide ligand acting at ARs, ρ-TIA, is a 19-residue toxin from *Conus tulipa*, and has been classified as a non-competitive α_1B_-AR antagonist that accelerates ^3^H-prazosin dissociation kinetics and antagonizes α_1B_-AR activation by an insurmountable mechanism [7]. A recent experimentally-based model shows that ρ-TIA interacts primarily with extracellular loop 3 (ec3) of the α_1B_-AR, consistent with its allosteric properties [18]. Thus, both MT7 and ρ-TIA display a negative allosteric mode of action by interacting with extracellular loops of their receptor targets, namely ec2 of the M1 AChR, and ec3 of the α_1B_-AR. ρ-TIA, however, shows only 10 to 25-fold selectivity for the α_1B_-AR over the other α_1_-AR subtypes, and has been described as a competitive antagonist at the α_1A_-AR although it does not fully inhibit ^125^I-HEAT binding [19].

These observations have led to hypotheses regarding the mode of action of these peptide toxins at receptor targets. The aims of our study were to use equilibrium and kinetic binding experiments to establish the pharmacological behavior of ρ-Da1a at the α_1A_-AR, to define the effect of ρ-Da1a on agonist-stimulated Ca^2+^ release, and to use site-directed mutagenesis to analyze the α_1A_-AR binding site for this peptide toxin.

## Experimental Procedures

^125^I-HEAT, ^3^H-prazosin, ^3^H-rauwolscine and ^3^H-CGP-12177 were purchased from PerkinElmer (Courtaboeuf, France). Non radioactive HEAT was obtained from Tocris (Ellisville, Missouri, USA), and 5-(N-ethyl-N-isopropyl-amiloride (EPA), prazosin, yohimbine, and propranonol were obtained from Sigma-Aldrich (St Quentin-Fallavier, France).

### Protein quantification

Total protein and membrane protein concentrations were determined using the Bio-Rad protein assay, with bovine serum albumin as standard.

### Site-directed mutagenesis

α_1A_-AR cDNA inserted in the prK5 vector was kindly provided by Michael Brownstein (Craig Venter Institute, Rockville, MD). Point mutations were introduced into the α_1A_-AR gene by sense and antisense primers (Sigma-Aldrich, St Quentin-Fallavier, France) containing the desired changes, using the QuikChange Site-Directed Mutagenesis kit. The incorporation of each mutation was verified by DNA sequencing. The variants F308^7.35^A and F312^7.39^A were generous gifts from Dr. Diane Perez (The Cleveland Clinic Foundation, Cleveland, Ohio, USA).

### Cell culture and membrane preparation

CHO cells stably expressing α_1_-ARs were kindly provided by Dr. Hervé Paris (INSERM U858, Toulouse, France) and were grown in a 50∶50 Dulbecco's Modified Eagle's Medium (DMEM)/Ham's F12 medium supplemented with 10% (v/v) foetal bovine serum (FBS), glutamine (2 mM), penicillin (100 units/ml) and streptomycin (100 µg/ml) at 37°C with 5% CO_2_. COS-7 cells were grown at 37°C under 5% CO_2_ in Dulbecco's modified Eagle's medium containing 10% fetal calf serum, 1% penicillin and 1% glutamine (Sigma-Aldrich, St Quentin-Fallavier, France). At 80% confluence, the cells were transfected using a calcium phosphate precipitation method for transient expression of the genetic construct. After 48 h incubation at 37°C, cells were harvested and the membranes were prepared as follow. Cells were washed with ice-cold phosphate buffer and centrifuged at 1700 g for 10 min (4°C). The pellet was suspended in ice-cold buffer (1 mM EDTA, 25 mM sodium phosphate, and 5 mM MgCl_2_, pH 7.4) and homogenized using an Potter-Elvehjem homogenizer (Fisher Scientific Labosi, Elancourt, France). The homogenate was centrifuged at 1700 g for 15 min (4°C). The sediment was resuspended in buffer, homogenized, and centrifuged at 1700 g for 15 min (4°C). The combined supernatants were centrifuged at 35,000 g for 30 min (4°C), and the pellet was suspended in the same buffer (0.1 ml/dish). The CHO cells used for Ca^2+^ release experiments also stably express the human α_1A_-AR (B_max_ 531±94 fmol/mg protein, pK_D_ for ^125^I-HEAT 9.2±0.09 [20]. Cells were grown in a 50∶50 Dulbecco's Modified Eagle's Medium (DMEM)/Ham's F12 medium supplemented with 10% (v/v) foetal bovine serum (FBS), glutamine (2 mM), penicillin (100 units/ml) and streptomycin (100 µg/ml) at 37°C with 5% CO_2_. Media was changed every 2–3 days and cells were passaged when confluent with 0.05% trypsin and 0.02% EDTA.

### Binding assays

We used ^3^H-prazosin and ^125^I-HEAT (all incubations were done in the dark) as selective ligands for α_1_-ARs, ^3^H-rauwolscine for α_2_-ARs and ^3^H-CCGP-12177 for β-ARs. Non-specific binding to α_1_, α_2_ and β-ARs was measured in presence of prazosin (10 µM), yohimbine (10 µM) and propanolol (10 µM), respectively. Binding experiments were performed in a 100 µL reaction mix at room temperature in buffer composed of 50 mM Tris-HCl, pH 7.4, 10 mM MgCl_2_, 1 g/L BSA. Reactions were stopped by filtration through 96 GF/C filter plates pre-incubated with 0.5% polyethylenimine. An aliquot of 25 µL of Microscint 0 was added onto each dry filter and the radioactivity was quantified on a TopCount beta counter with a 33% yield (PerkinElmer, Courtaboeuf, France). Saturation binding assays were performed using a fixed amount of receptors and a series of concentrations of ^125^I-HEAT with an incubation time of 1 h. Competition binding assays were performed by mixing the radioligand (2 nM of ^3^H-prazosin or ^3^H-rauwolscine, 0.2–1.3 nM of ^125^I-HEAT, 6 nM of ^3^H-CGP-12177) with a range of competitor concentrations before adding membranes (α_1A_-AR: 1 µg for ^3^H-prazosin and 0.1 µg for ^125^I-HEAT, α_1B_: 3 µg, α_1D_: 29 µg, α_2A_: 140 µg; α_2B_: 100 µg, α_2C_: 3 µg, β_1_: 3 µg, β_2_: 1.5 µg, or α_1A_-mutants: 0.1–1 µg), for 16 h of incubation. Dissociation kinetics experiments were performed by pre-equilibrating ^125^I-HEAT (400 pM) or ^3^H-prazosin (2 nM) for 3 hours with α_1A_-AR COS-7 cell membranes (0.2 or 1 µg, respectively). Radiotracer dissociation was then measured following addition of HEAT (5 µM) or prazosin (10 µM) alone or with ρ-Da1a (2.5 µM), 5-(N-ethyl-N-isopropyl)-amiloride (EPA, 150 µM) or adrenaline (2 mM).

### Measurement of intracellular Ca^2+^ concentration

CHO-K1 cells expressing the α_1A_-AR were seeded at 2×10^4^ cells per well in 96-well plates overnight. The following morning, the media was removed and cells washed three times in a modified Hanks' buffered saline solution (HBSS; composition in mM: NaCl 150, KCl 2.6, MgCl_2_.2H_2_O 1.18, D-glucose 10, Hepes 10, CaCl_2_.2H_2_O 2.2, probenecid 2, pH 7.4) containing BSA 0.5% (w/v). In light-diminished conditions cells were treated with fluoro-4 (0.1% v/v in modified HBSS, 1 h, 37°C). Excess fluoro-4 not taken up by the cells was removed by washing twice in modified HBSS and then cells incubated for a further 30 min in the absence or presence of differing concentrations of ρ-Da1a before the assay plate was transferred to a FlexStation (Molecular Devices, Palo Alto CA, USA). Real-time fluorescence measurements were recorded every 1.7 seconds over 200 seconds, with agonist (noradrenaline, phenylephrine, A61603 or oxymetazoline) additions occurring after 17 seconds, using an excitation wavelength of 485 nm and reading emission wavelength of 520 nm. All experiments were performed in duplicate. Agonist responses represent the difference between basal fluorescence and peak [Ca^2+^]i measurements expressed as a percentage of the response to A23187 (1 µM) in each experiment.

### Data analysis

Binding data were analyzed by nonlinear regression using the KaleidaGraph 4.0 software (Synergy software, Reading, PA). pK_D_ values and Bmax (number of binding sites) were determined by applying a nonlinear regression to data obtained with saturation binding assays. The nonlinear regression used was BS = (Bmax*A)/K_D_+A, where BS is the specific binding, Bmax the number of binding sites, A the concentration of radioligand, and K_D_ the dissociation constant of the radioligand. Data resulting from competition binding assays were analyzed using the Hill equation for IC_50_ and curve slope estimations. The binding affinity (pK_i_) of ρ-Da1a was determined from the IC_50_ value of inhibition curves using the Cheng and Prussof equation [21]. The linear curves were analyzed with IC_50_ = K_i_+(L/K_D_)*K_i_. Dissociation kinetics were analyzed using a simple equation of exponential decay BS * exp (-K_off_*t), where BS is the specific binding at time zero and K_off_ is the dissociation rate constant. Results are expressed as mean ± s.e. mean from n independent experiments. One-way Anova test was used to compare values. A p<0.05 was accepted for statistical significance.

Values for intracellular Ca^2+^ release are expressed as mean ± s.e. mean from n independent experiments. Data were analysed using non-linear curve fitting (Graph Pad PRISM v5.02) to obtain pEC_15_ values for the [Ca^2+^]i assays. Antagonists such as ρ-Da1a that have slow dissociation kinetics are prone to display hemi-equilibrium artifacts in functional transient responses such as measurement of intracellular Ca^2+^ levels. As such, when competing with an agonist, the maximal response achieved by the agonist reduces in the presence of higher antagonist concentrations due to the inaccessibility of a large pool of the receptors in the time taken for the transient response to occur [22]. This affects the ability of a Schild analysis to estimate the pK_B_ of ρ-Da1a. In order to account for this, the pK_B_ value for ρ-Da1a was calculated by the modified Lew-Angus method [23] using pEC_15_ values, based on the extent of reduction in agonist maximal responses in the presence of ρ-Da1a. The pEC_15_ values were plotted against the concentration of antagonist and non-linear regression applied [23], [24] to estimate pK_B_ values for ρ-Da1a against each of the four different agonists.

### Homology modeling

A model of the α_1A_-AR was generated with MODELLER [25]. The receptor with the most similar sequence to the α_1A_-AR is the β_2_-AR subtype, with an overall amino acid sequence identity of 21% [26], [27], identity within 7TM domain from helix 1 to helix 8 (excluding intracellular ic3 loop) 38%, sequence similarity 61% [BLASTP]. Nine β_2_-AR structures are available (2RH1, 3D4S, 3KJ6, 3NY8, 3NY9, 3NYA, 3PDS, 3P0G, 3SN6) and are very similar (Cα RMSDs<1.5 Å for 253 residues). We used the X-ray structure with the highest resolution (2RH1) as a template [28].

## Results

ρ-Da1a, (previously AdTx1) [9], was renamed according to a rational nomenclature [29]. A recombinant expression system producing the toxin with an extra glycine residue at its N-terminus was developed (Figure S1 in File S1). Recombinant ρ-Da1a displays the same affinity as the chemically synthesized toxin, indicating that the N-terminal glycine has no consequences for function. The pharmacological experiments reported in this study were performed with the recombinant form of the toxin.

### Selectivity of ρ-Da1a

**Figure 1:**
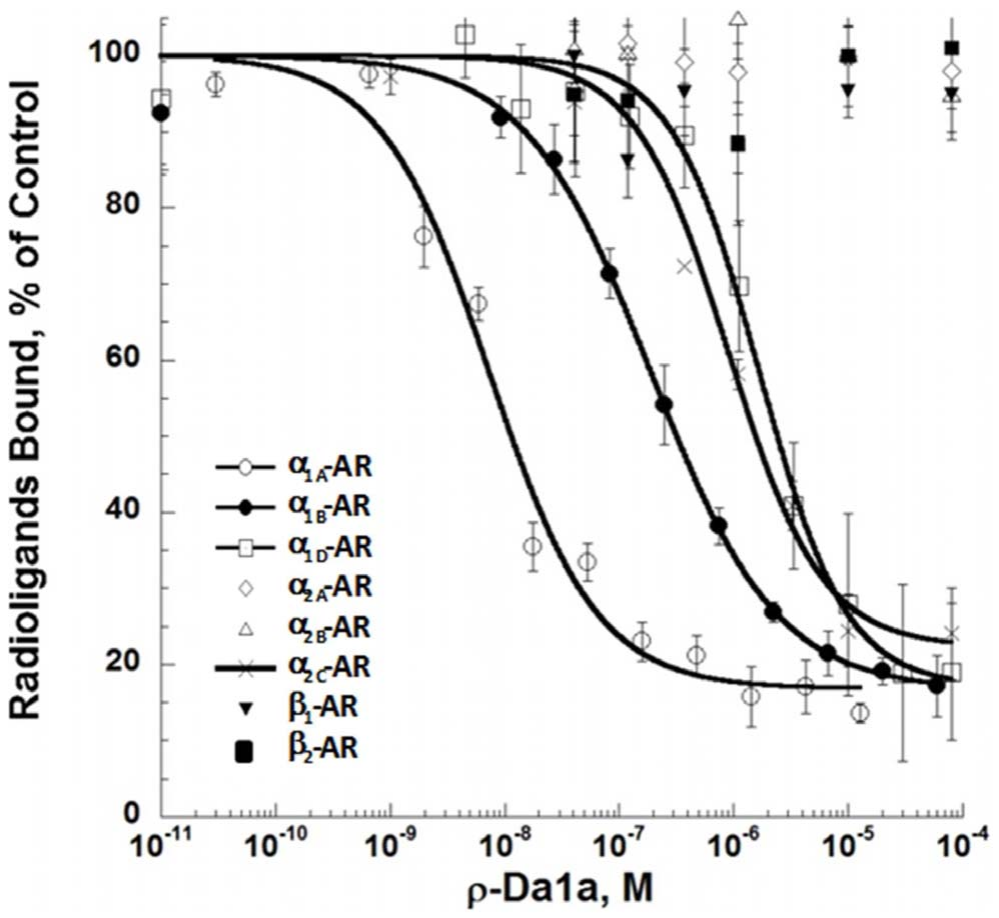
Pharmacological profile of ρ-Da1a binding to various human AR subtypes expressed in eukaryotic cells. Binding inhibition curves for ^3^H-prazosin (2 nM), ^3^H-rauwolscine (2 nM) and ^3^H-CGP-12177 (6 nM) on hα_1A_- (1 µg, ○), hα_1B_- (3 µg, •), hα_1D_- (29 µg, □), hα_2A_- (140 µg, ◊), hα_2B_- (100 µg, Δ), hα_2C_- (3 µg, x), β_1_- (3 µg,▾) and β_2_-AR (1.5 µg, ▪) with recombinant ρ-Da1a. n = 4.

ρ-Da1a affinity was recently determined in tissue preparations, and using human and rat α_1_-ARs expressed in yeast [9]. To complete the ρ-Da1a selectivity profile, we expressed human ARs in eukaryotic cells and performed competition binding with additional receptor subtypes. The pKi values derived from these experiments [21] were: 9.19±0.09 for α_1A_-ARs, 7.28±0.09 for α_1B_-ARs, 6.85±0.08 nM for α_2C_-ARs, and 5.95±0.08 for α_1D_-ARs. No significant effect was observed with 10 µM of ρ-Da1a at α_2A_, α_2B_, β_1_, or β_2_-ARs. For α_1A_- and α_1B_-ARs, even the highest concentrations of ρ-Da1a did not completely inhibit ^3^H-prazosin binding, which remained at 18±3% and 17±1%, respectively (Fig. 1).

### ρ-Da1a, prazosin and HEAT compete at α_1A_-ARs

**Figure 2:**
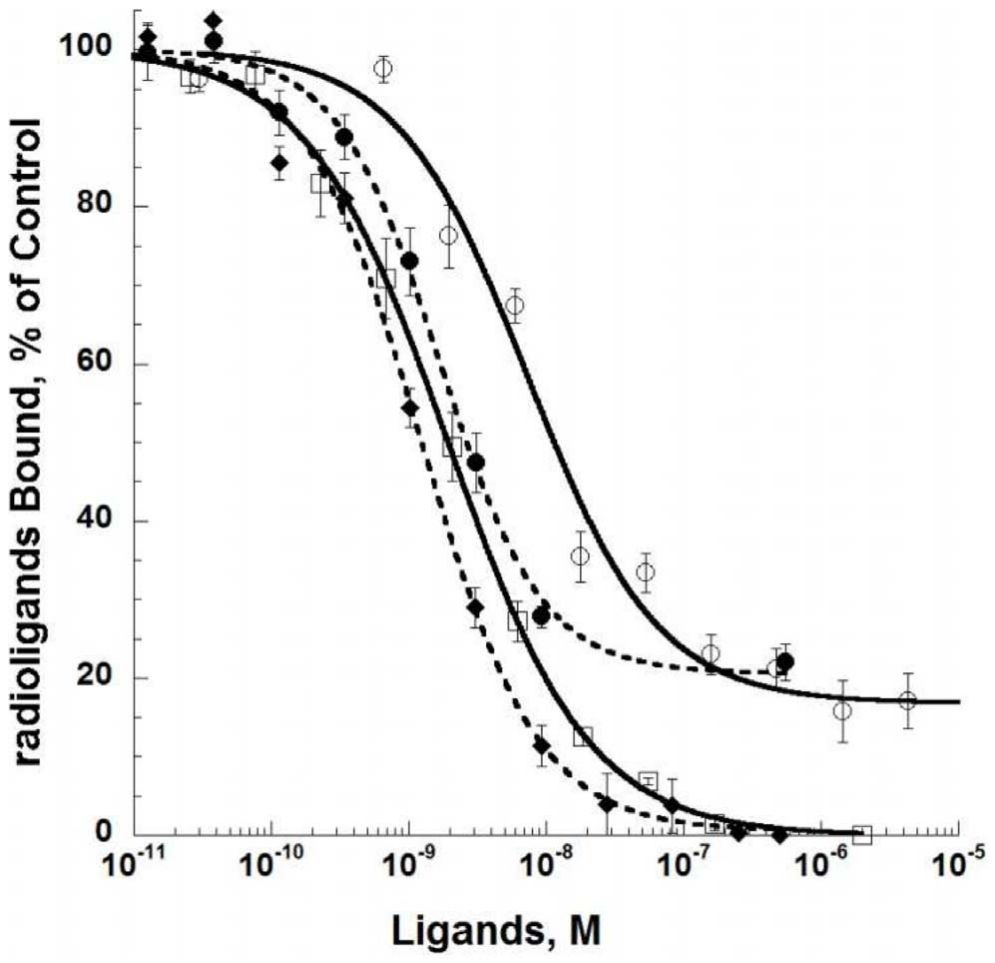
Inhibition of ^3^H-prazosin (2 nM, 1 µg, open symbols) by HEAT (□), and ρ-Da1a (circle), and inhibition of ^125^I-HEAT (0.2 nM, 0.2 µg, full symbols) binding by prazosin (♦) and ρ-Da1a (circle) to α_1A_-AR. n = 3.

^3^H-prazosin binding was fully inhibited by HEAT (pKi 9.66±0.08 nM, Hill slope 0.85, Fig. 2) and ^125^I-HEAT binding was fully displaced by prazosin (pKi 9.18±0.07 nM, Hill slope 0.95). However, as observed with ^3^H-prazosin, ρ-Da1a interacts very efficiently with the α_1A_-AR (pKi 9.26±0.07 nM, Hill slope 0.92, Fig. 2), but does not inhibit more than 80% of ^125^I-HEAT binding. This residual binding is stable with time, as we detected no variation with incubation times from 2 to 24 hours (data not shown).

**Figure 3:**
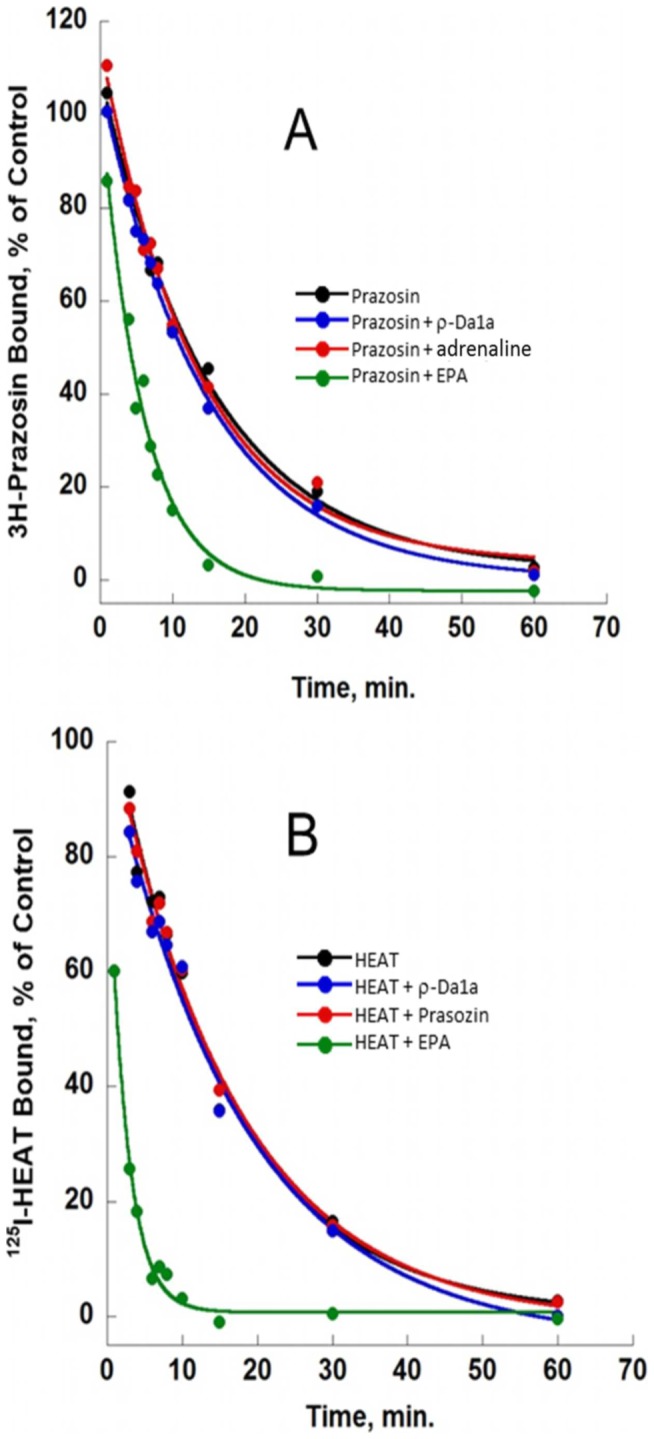
Influence of various ligands on ^3^H-prazosin and ^125^I-HEAT dissociation. Panel A: Dissociation of ^3^H-prazosin (2 nM) binding to α_1A_-AR (1 µg) in the presence of prazosin (10 µM, black), prazosin plus ρ-Da1a (2.5 µM, blue), prazosin plus adrenaline (2 mM, red) and prazosin plus EPA (150 µM, green). Panel B : dissociation of ^125^I-HEAT (0.4 nM) binding to α_1A_-AR (0.2 µg) in the presence of HEAT (5 µM, black), HEAT plus ρ-Da1a (2.5 µM, blue), HEAT plus prazosin (10 µM, red) and HEAT plus EPA (150 µM, green). n = 2.

Dissociation kinetic experiments are classically used to identify negative allosteric modulators [30], [31]. The influence of ρ-Da1a on the dissociation kinetics of ^3^H-prazosin and ^125^I-HEAT was studied in comparison with adrenaline and the negative allosteric modulator EPA (Fig. 3). ^3^H-prazosin dissociation from α_1A_-ARs was mono-exponential and the dissociation rate was 0.05±0.01 min^−1^. Consistent with previous studies [32], this value was increased 2.6 times in the presence of 150 µM EPA (K_off+ EPA_ = 0.15 min^−1^). In contrast, neither 2 mM adrenaline nor 2.5 µM ρ-Da1a affected the ^3^H-prazosin dissociation rate (K_off+adrenaline_ = 0.054 min^−1^; K_off+ρ-Da1a_ = 0.059 min^−1^, Fig. 3, n = 2) in the presence of excess prazosin. ^125^I-HEAT dissociation rates were measured in the absence (K_off_ = 0.062 min^−1^) and in the presence of ρ-Da1a (K_off HEAT+ρ-Da1a_ = 0.058 min^−1^), prazosin (K_off HEAT+prazosin_ = 0.06 min^−1^), and EPA (K_off HEAT+EPA_ = 0.37 min^−1^, Fig. 3, n = 2): neither prazosin nor ρ-Da1a affected the HEAT dissociation rate; whereas EPA increased the dissociation rate by six-fold.

**Figure 4:**
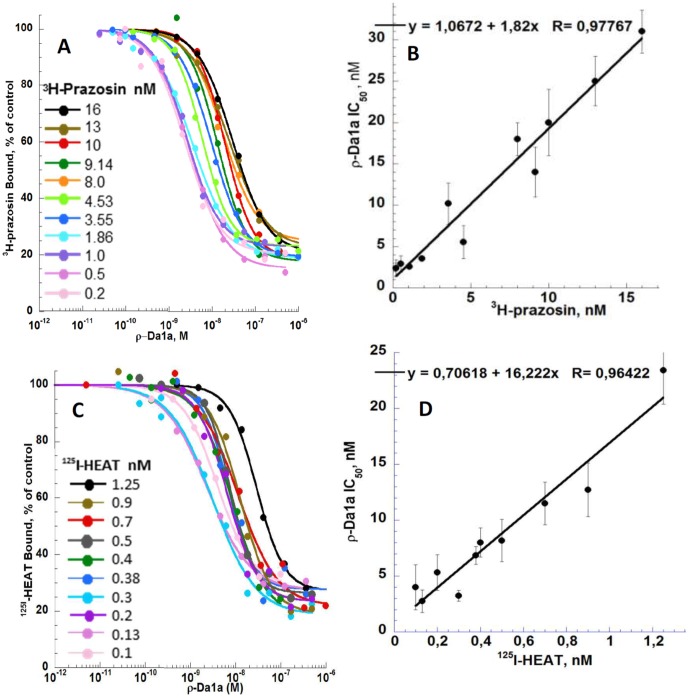
Inhibition of the binding of a series of concentrations of ^3^H-prazosin and ^125^I-HEAT to α_1A_-AR by ρ-Da1a. Panel A ^3^H-prazosin binding (from 0.2 to 16 nM) inhibited by ρ-Da1a. Panel B ^125^I-HEAT binding (from 0.1 to 1.25 nM) inhibited by ρ-Da1a. Panel C and D: Fitting, by the Cheng and Prusoff equation IC_50_ = Ki+Ki(L/Kd), of IC_50_ values as a function of the radiotracer concentrations.

The ρ-Da1a IC_50_ values were determined using various concentrations of radiotracers in competition binding experiments (Fig. 4). Eleven concentrations of ^3^H-prazosin (0.2, 0.5, 1.0, 1.86, 3.55, 4.53, 8.0, 9.14, 10, 13 and 16 nM) were dose-dependently inhibited by ρ-Da1a (IC_50_ of 2.4, 2.9, 2.6, 3.55, 10.2, 5.54, 18, 14, 20, 25 and 31 nM) with Hill slopes between 0.8 and 1.1. Residual binding in the presence of ^3^H-prazosin fluctuated between 15 to 25% of the total binding, but did not show any trend to concentration-dependence. The curve IC_50ρ-Da1a_ as a function of ^3^H-prazosin concentration (L) fitted the linear regression IC_50ρ-Da1a_ = 1.067+1.82*L, incompatible with a negative allosteric modulation (Fig. 4A). Using the equation IC_50ρ-Da1a_ = Ki_ρ-Da1a_+(Ki_ρ-Da1a_ * L_prazosin_/Kd_prazosin_) [21], this experiment gave a Ki_ρ-Da1a_ of 1.067 nM (pKi 8.97) and a Kd_prazosin_ of 0.586 nM (pKd 9.23). An analogous experiment was performed using ^125^I-HEAT. Ten concentrations (0.1, 0.13, 0.2, 0.3, 0.38, 0.4, 0.5, 0.7, 0.9 and 1.25 nM) were inhibited by ρ-Da1a (IC_50_ of 4.0, 2.75, 5.3, 3.23, 6.84, 8.0, 8.18, 11.5, 12.7 and 23.4 nM) with Hill slopes between 0.9 and 1.4. Residual binding fluctuated between 18 to 28% of the total binding (Fig. 4B). IC_50ρ-Da1a_ as a function of ^125^I-HEAT concentrations fitted the equation IC_50ρ-Da1a_ = 0.706+16.22*L which gave a Ki_ρ-Da1a_ of 0.706 nM (pKi 9.15) and a Kd_HEAT_ of 0.0435 nM (pKd 10.36).

### Insurmountable antagonism of intracellular Ca^2+^ release by ρ-Da1a

**Figure 5:**
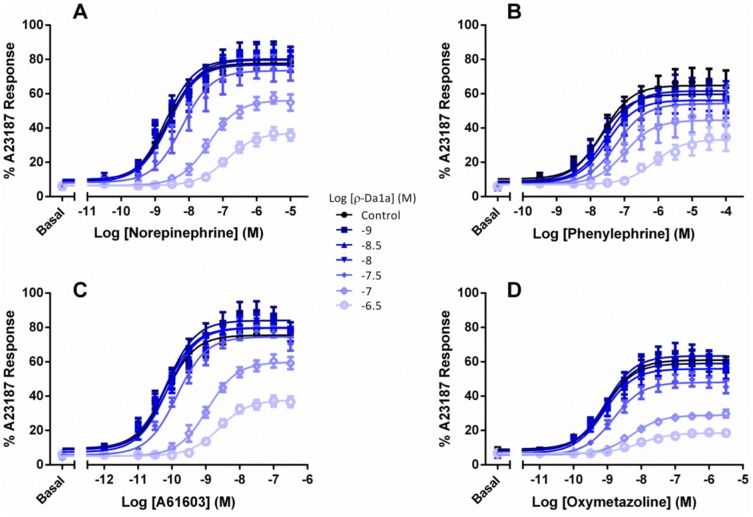
Concentration-response curves for stimulation of Ca^2+^ release by the α_1A_-AR. Agonist responses represent the difference between basal fluorescence and the peak [Ca^2+^]i (reached within 20 sec of agonist addition), expressed as a percentage of the response to the Ca^2+^ ionophore A23187 (1 µM). Concentration-dependent Ca^2+^ release was stimulated by noradrenaline (panel A), phenylephrine (panel B), A61603 (panel C) or oxymetazoline (panel D). Concentration response curves were performed in the presence or absence of differing concentrations of ρ-Da1a (• control, ▪ 1 nM, ▴ 3 nM, ▾ 10 nM, ♦ 30 nM, ◊ 100 nM, ○ 300 nM). Values are means ± SEM of 3–4 independent experiments.

We next tested the effect of ρ-Da1a on responses to noradrenaline and phenylephrine (phenethylamine agonists) and A61603 and oxymetazoline (imidazoline agonists). In CHO-K1 cells expressing the α_1A_-AR, all agonists stimulated Ca^2+^ release (Figure 5), with pEC_50_ and E_max_ values consistent with previous work [20] – noradrenaline pEC_50_ 8.63±0.08, E_max_ (as a percentage of peak A23187 response) 77.8±1.5, phenylephrine pEC_50_ 7.64±0.16, E_max_ 64.8±2.4, A61603 pEC_50_ 10.17±0.07, E_max_ 75.5±1.3, and oxymetazoline pEC_50_ 9.09±0.08, E_max_ 60.8±1.3. Increasing concentrations of ρ-Da1a reduced the maximal response to each of the four agonists while shifting the curves to the right. The pK_B_ values for ρ-Da1a were calculated by a modified Lew-Angus method [23] and were similar irrespective of the agonist employed (7.71±0.05 vs noradrenaline; 7.60±0.04 vs phenylephrine; 7.66±0.10 vs A61603; 7.67±0.05 vs oxymetazoline).

### Molecular characterization of the ρ-Da1a/α_1A_-AR interaction

**Figure 6:**
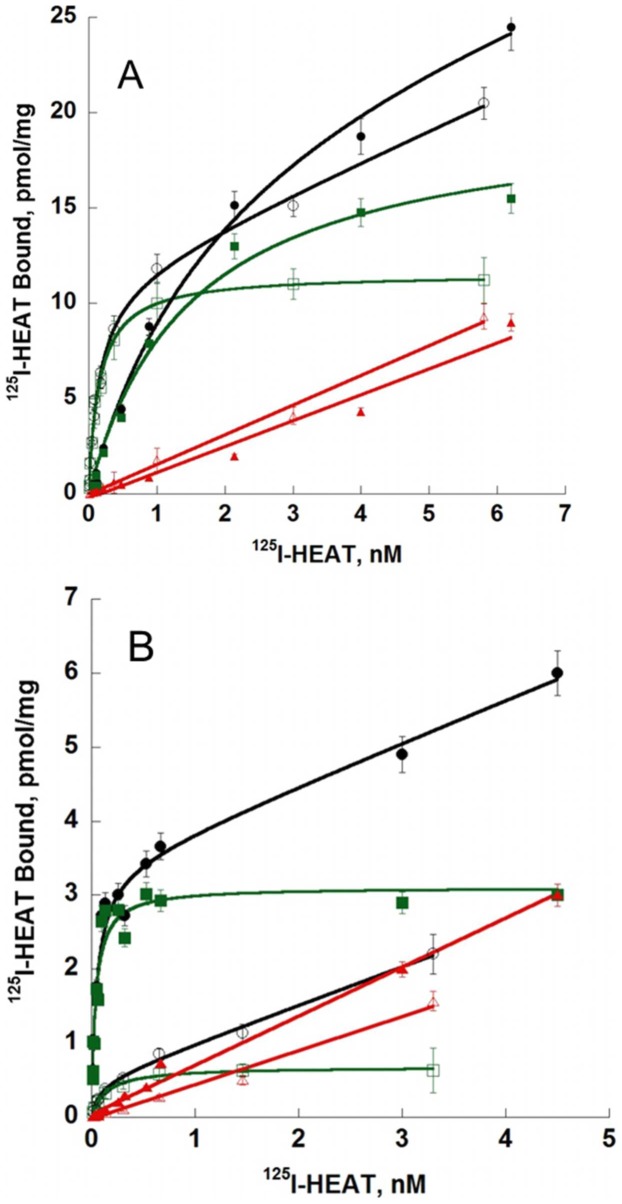
Saturation experiments with ^125^I-HEAT on receptor variants.

We investigated the involvement of residues within the orthosteric pocket of α_1_-ARs that are known to interact with agonists and/or antagonists: F86^2.64^ [33], D106^3.32^ (D125 in α_1B_-AR) [34]–[36], F187^5.41^ [37], S188^5.42^ and S192^5.46^ [38], F288^6.51^ (F310 on α_1B_-AR) [18], [39], M292^6.55^ [40] and F308^7.35^ and F312^7.39^ [18], [41] (superscripts refer to the Ballesteros-Weinstein numbering system for residues in 7TM helices). In addition, we tested the positions F193^5.47^ and F281^6.44^, predicted to play a role in the stabilization of the active state of α_1A_-AR [42] (Table 1). In saturation binding experiments, ^125^I-HEAT affinity values for D106^3.32^A, F193^5.47^A, F281^6.44^A, F288^6.51^A, M292^6.55^A and F308^7.35^A were not significantly different from the wild type receptor. One mutated receptor, F187^5.41^A, had significantly higher affinity for ^125^I-HEAT with a pK_D_ of 10.70±0.005 compared to wild type pK_D_ 10.05±0.09 (Table 1, p<0.05). Only the F86^2.64^A mutant showed a substantial 23-fold loss of ^125^I-HEAT affinity (pK_D_ 8.68±0.09, p<0.05, Fig. 6, Table 1). The transiently transfected mutant receptors showed marked variability in expression level, with B_max_ values ranging from 0.63 up to 29 pmol/mg protein compared to 11.3 pmol/mg protein for the wild type α_1A_-AR (Table 1), however there was no correlation between receptor abundance and the observed binding affinity of ^125^I-HEAT. For example, D106^3.32^A (0.63 pmol/mg protein) and F308^7.35^A (29 pmol/mg protein) both displayed a similar pKd to each other and to the wild type receptor.

**Table 1:** Effect of human α_1A_-AR mutations on receptor expression and affinity for HEAT and ρ-Da1a.

Variant	Position	^125^I- HEAT			HEAT		ρ-Da1a	
		Bmax pmol/mg	pK_d_	Ratio	pKi	ratio	pKi	Ratio
WT		11.3±2.3	10.05±0.14	1	9.57±0.08	1	9.26±0.07	1
F86A	2.64	22.6±2.4	8.68±0.09	**23** *	8.21±0.09	**23** *	7.70±0.06	**36** *
D106A	3.32	0.63±0.05	9.82±0.11	1.7	9.74±0.12	0.67	8.48±0.11	**6.0** *
F187A	5.41	20.5±3.5	10.70±0.01	**0.22** *			9.12±0.07	1.4
SS-AA	5.42–5.46	13.3±3.3	10.15±0.01	0.78			8.38±0.09	**7.6** *
F193A	5.47	11.5±2.2	9,60±0.09	2.8			9.64±0.11	0.42
F281A	6.44	18.2±4.8	9.46±0.09	3.9			9.66±0.09	0.40
F288A	6.51	14.3±2.5	9.92±0.12	1.3			8.00±0.08	**18** *
M292A	6.55	15.8±3.5	9.69±0.09	2.2			9.41±0.11	0.71
F308A	7.35	29±4.2	9.66±0.11	2.4			9.15±0.07	1.3
F312A	7.39	3.2±0.12	10.40±0.10	0.44			7.28±0.10	**93** *

* for p<0.05. Position refers to the Ballesteros-Weinstein numbering scheme for residues within TM domains of G protein-coupled receptors. n = 3–6.

**Figure 7:**
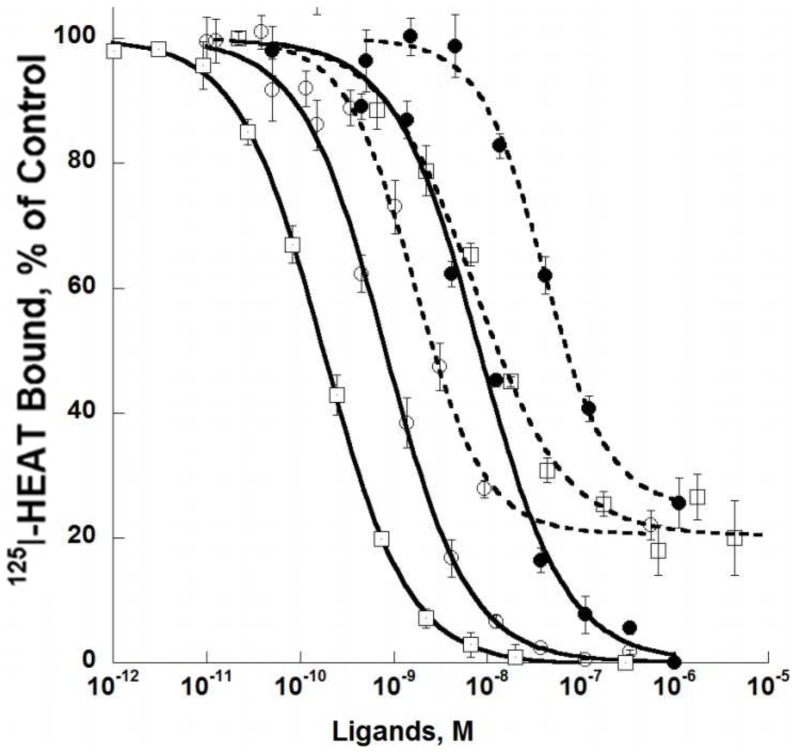
Receptor affinities for ρ-Da1a (dash lines) and HEAT (solid lines) on mutated α_1A_-ARs. Binding inhibition curves for ^125^I-HEAT binding to WT (200 pM, 0.2 µg, ○), D106^3.32^A (200 pM, 1 µg, □) and F86^2.64^A (1.3 nM, 0.8 µg, •) receptor variants. n = 3–4.

Curves for competition of HEAT and ρ-Da1a with binding of ^125^I-HEAT are shown at wild type, D106^3.32^A and F86^2.64^A receptors (Figure 7). As seen in the saturation binding experiment, HEAT had similar affinity for the D106^3.32^A variant (pKi 9.74±0.12) and the wild type receptor (pKi 9.57±0.08) but was strongly affected by the F86^2.64^A mutation (pKi 8.21±0.09). ρ-Da1a affinity at the D106^3.32^A variant was reduced by 6-fold (pKi 8.48±0.11) compared to the wild type receptor while affinity at F86^2.64^A was reduced by 36-fold (pKi 7.70±0.06). The mutation F86^2.64^A affects both HEAT and ρ-Da1a affinities, suggesting that the structural organization of the receptor could have been perturbed by this modification. We used the radioligand ^3^H-prazosin to examine this point and found that prazosin affinity for the F86^2.64^A mutant (pKd 9.21±0.07) was very close to the one measured for wild type receptor (9.26±0.05; data not shown). While this mutation may alter interactions between F86^2.64^ and other aromatic residues within the orthosteric pocket [43], the retention of prazosin affinity indicates that there is no global effect on the native structural conformation of the receptor. As seen for the wild type α_1A_-AR, ρ-Da1a inhibited only 75–80% of ^125^I-HEAT binding in experiments with these mutant receptors.

**Figure 8:**
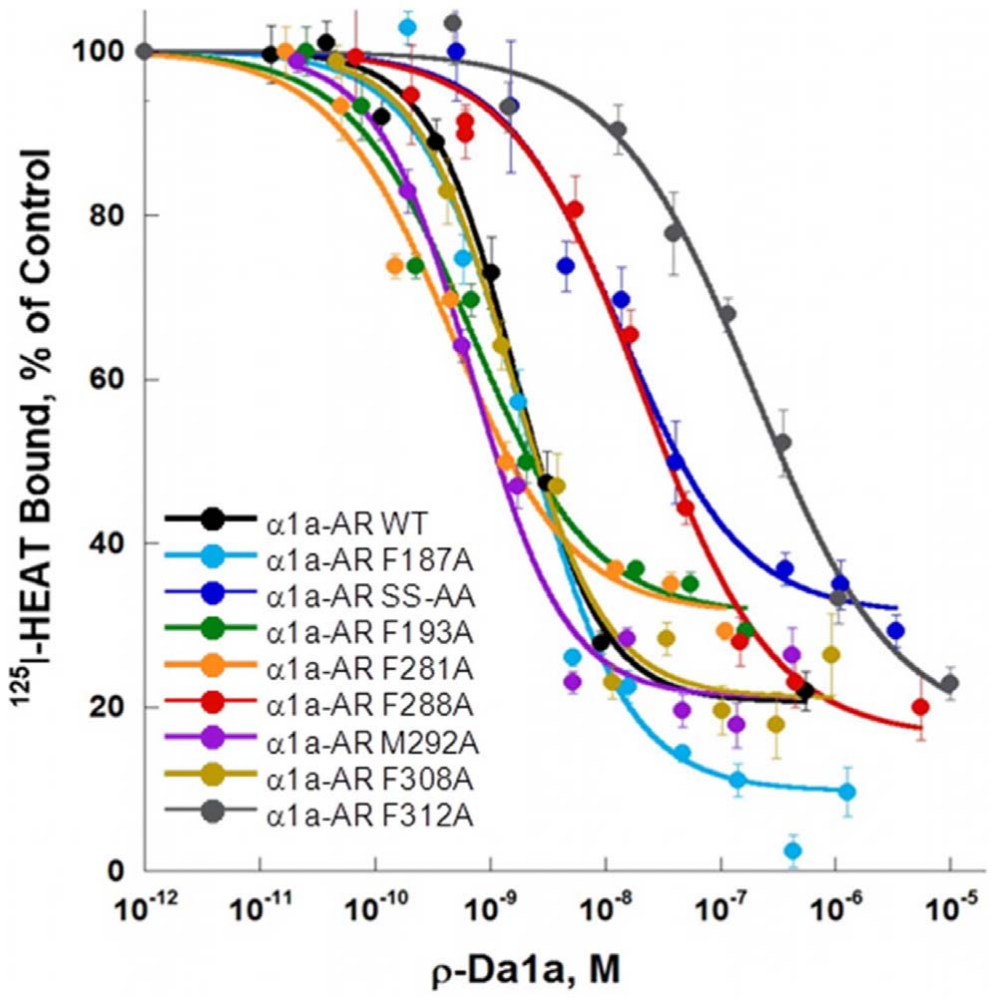
Receptor affinities for ρ-Da1a on mutated α_1A_-ARs. Binding inhibition curves for ^125^I-HEAT (200 pM) binding to WT (0.2 µg, black), F187^5.41^A (0.15 µg, light blue), the double S188^5.42^,S192^5.46^-AA (0.3 µg, dark blue), F193^5.47^A (0.25 µg, green), F281^6.44^A (0.15 µg, orange), F288^6.51^A (0.2 µg, red), M292^6.55^A (0.2 µg, purple), F308^7.35^A (0.1 µg, brown), F312^7.39^A (0.8 µg, grey), n = 3–4.

ρ-Da1a affinities were tested on eight additional receptor variants (Fig. 8). At the F187^5.41^A, M292^6.55^A and F308^7.35^A variants, ρ-Da1a inhibited ^125^I-HEAT binding with affinities similar to the wild type (Table 1). However the F288^6.51^A and the F312^7.39^A variants decreased ρ-Da1a affinity by 18 and 93 times, with pKi of 8.00±0.08 and 7.28±0.10, respectively. Again, ρ-Da1a left residual binding between 20 and 30%, except for the F187^5.41^A variant (9±3%) receptor (Fig. 8).

## Discussion

ρ-Da1a is the first natural peptide shown to be selective for the α_1A_-AR. Due to its high selectivity and potent relaxing effect on isolated prostate smooth muscle [5], [9], [44], the peptide is in the process of therapeutic development. In recombinant expression systems, ρ-Da1a can be produced with a final yield of 5 mg per liter of culture. The recombinant toxin interacts with the α_1A_-AR in a similar manner to the chemically synthesized one.

The selectivity profile of ρ-Da1a for human ARs was established, and confirmed a sub-nanomolar affinity for the α_1A_-AR subtype: the order of selectivity is α_1A_>α_1B_>α_2C_>α_1D_>>α_2A_ = α_2B_ = β_1_ = β_2_. The affinities of ρ-Da1a for α_1_-ARs expressed in yeast or in mammalian cells were slightly different: 0.35 and 0.55 nM for α_1A_-AR, 420 nM and 1110 nM for α_1D_-AR, and 317 and 53 nM for α_1B_-AR respectively. These differences in toxin affinity may be related to differences in associated lipids or proteins in the membranes of these cells, as described for the μ-opioid [45] or the dopamine D_2S_ receptors [46].

We have characterized the interaction between ρ-Da1a and the α_1A_-AR by a series of binding and functional experiments. Our findings from Ca^2+^ release assays, competition binding and radioligand dissociation curves in the presence of ρ-Da1a generally indicate competition between the toxin and small molecule ligands. On the other hand, ρ-Da1a was unable to completely inhibit orthosteric radioligand binding to α_1A_-ARs regardless of the time of incubation (2 to 24 h), the radioligand (^3^H-prazosin or ^125^I-HEAT), or the expression system (yeast, CHO, or COS-7 cells), a finding more consistent with non-competitive interaction. To examine functional antagonism by ρ-Da1a, we measured blockade of intracellular Ca^2+^ release following 30 min pre-incubation of CHO-α_1A_-AR cells with the toxin (Fig. 5). The observed insurmountable antagonism indicates that at higher concentrations of ρ-Da1a, a large proportion of receptors are inaccessible to agonist during the time taken for the transient Ca^2+^ response [22]. This effect is in part due to the slow dissociation kinetics of ρ-Da1a [9], which prevents the system from reaching equilibrium under the assay conditions [22]. The reduction in E_max_ is governed by the efficacy of each agonist – for example oxymetazoline is a high affinity, low efficacy agonist that displays a greater loss of maximal response in the presence of ρ-Da1a than high efficacy agonists such as noradrenaline and A61603. Essentially there is lower receptor reserve for responses to oxymetazoline than to noradrenaline or A61603. Despite these differences in reduction of E_max_, the pK_B_ values for ρ-Da1a blockade of Ca^2+^ release remained the same irrespective of the agonist used (between 7.6 and 7.71). These data conform to “non-permissive” antagonism, where receptor occupancy by the toxin prevents simultaneous orthosteric agonist interaction [47]. In the converse situation where a toxin (for example MT7) binds to a receptor at a site distinct from the orthosteric pocket, receptors are able to bind simultaneously both the toxin and an agonist – illustrating “permissive” antagonism characteristic of allosteric modulators [48]. As different agonists adopt distinct poses in the orthosteric binding site, they have the capacity to differentially affect the affinity of an allosteric modulator for the receptor, thus pK_B_ values of the modulator are altered depending on the agonist used [48], [49]. Our finding that the pK_B_ of ρ-Da1a is the same for four agonists belonging to two distinct structural classes, and known to display signaling bias at the α_1A_-AR [20], corroborates our other data showing that ρ-Da1a has no effect on the dissociation rate of either ^3^H-prazosin or ^125^I-HEAT, and that ρ-Da1a affinity for the α_1A_-AR is reduced by mutation of residues within the orthosteric pocket.

Allosteric modulators are generally characterized by effects on the dissociation rate of orthosteric radioligands in kinetic binding experiments. For example, MT7 significantly affects the dissociation kinetics of ^3^H-N-methylscopolamine and ^3^H-acetylcholine in membranes expressing the M1 AChR [14], [16], and the negative allosteric modulator EPA (5-(N-ethyl-N-isopropyl-amiloride) substantially increases the dissociation rate of both ^3^H-prazosin and ^125^I-HEAT from the α_1A_-AR (Fig. 3 in this study, [32]). In contrast, ρ-Da1a has no effect on the dissociation rate of ^3^H-prazosin or ^125^I-HEAT (Fig. 3). Reciprocally, prazosin has no effect on the ^125^I-ρDa1a dissociation rate [9], indicating competitive behavior. In equilibrium binding experiments, there was a linear relationship between the IC_50_ of ρ-Da1a and the concentration of ^3^H-prazosin or ^125^I-HEAT (Fig. 4, panel C and D), again consistent with competitive behavior [50].

The observed competition between ρ-Da1a and radioligands at the α_1A_-AR suggested that the toxin directly interacts with the orthosteric binding pocket. To provide further evidence for this proposal, ten positions belonging to the orthosteric pocket of the α_1A_-AR were tested for effects on ρ-Da1a affinity. Previous studies on the α_1A_-AR have shown that residues F187^5.41^ [37] and M292^6.55^ [40] are important for agonist and/or antagonist binding, and the two residues F193^5.47^ and F281^6.44^ [42] participate in stabilizing the active conformation of the receptor. Aside from an increase of 4.5 fold in HEAT affinity at the F187^5.41^A variant, none of these mutations affected either ρ-Da1a or HEAT affinity (Table 1).

The negative charge of D106^3.32^ is expected to interact with the positive charge of biogenic amines [51], and no binding of ^3^H-prazosin to the α_1A_-AR variants D106^3.32^A or D106^3.32^A/N167F is observed [36]. The homologous D125^3.32^A variant of the α_1B_-AR has been expressed but showed no change in affinity for HEAT [52], although another study has shown a total loss of affinity for HEAT [34]. In our study, HEAT affinity was not affected by alanine substitution of residue D106^3.32^, whereas toxin affinity was reduced six-fold. The TM5 residues S188^5.42^/S192^5.46^ are one helical turn apart, and have been implicated in agonist binding and receptor activation. While either single mutation, S188^5.42^A or S192^5.46^A does not alter agonist binding, the double mutation reduces agonist affinity [38]. In our hands, the double mutation moderately reduced ρ-Da1a affinity by 7.6-fold.

We found three key mutations that have major importance for ρ-Da1a binding: F86^2.64^A, F288^6.51^A and F312^7.39^A. Residue F312^7.39^ in the α_1A_-AR has been described to interacts with prazosin and imidazoline-type agonists [41]. Phenylalanine F310 at position 6.51 in the α_1B_-AR (F288 in the α_1A_-AR) is a major determinant for the interaction with the aromatic ring of catecholamines and with α_1_-AR antagonists like prazosin and phentolamine [18], [39]. Thus, ρ-Da1a shares two major interaction points with prazosin, F288^6.51^ and F312^7.39^, and one with phenethylamine-type (F288^6.51^) and imidazoline-type (F312^7.39^) agonists. In addition, F86^2.64^ is the only residue unique to the α_1A_-AR subtype that is important for toxin affinity. In α_1B_-, α_2C_- and α_1D_-ARs, this position is occupied by a leucine, an asparagine and a methionine, respectively. F86^2.64^ was previously identified as a determinant for interaction of the α_1A_-AR with various antagonists [33]. While a F86^2.64^M receptor mutant did not show any changes in HEAT affinity [33], the F86^2.64^A one strongly decreased it while having no effect on prazosin affinity. This residue most likely contributes to the selectivity of ρ-Da1a for the α_1A_-AR subtype.

**Figure 9:**
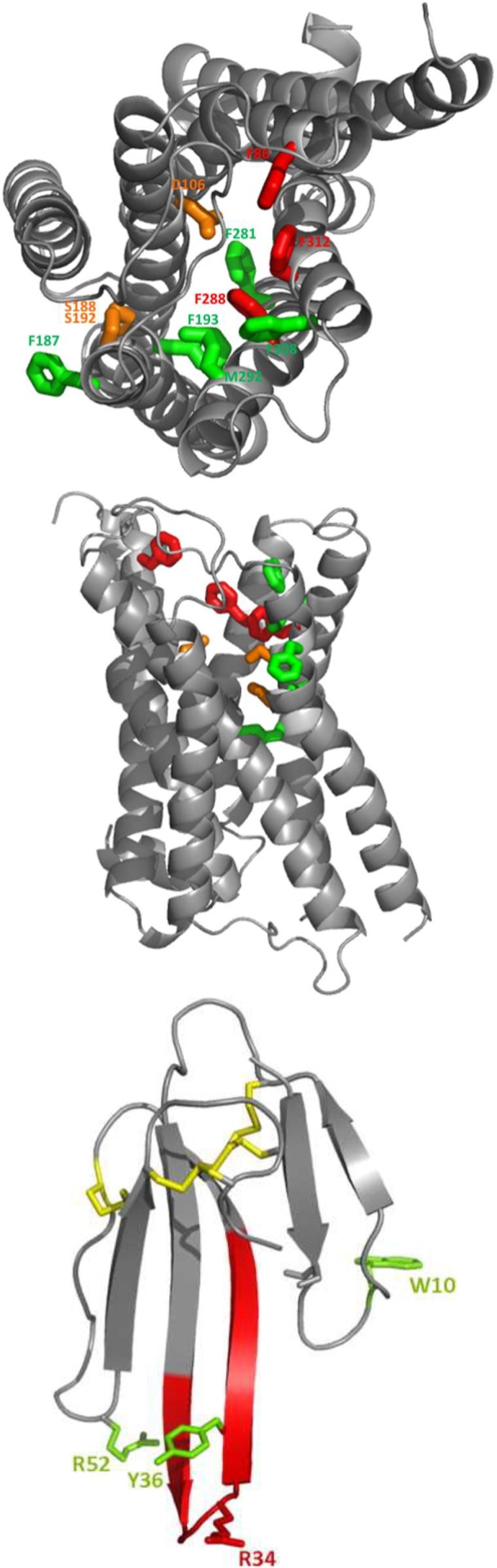
Homology modelling of the ρ-Da1a binding site in the α_1A_-AR and the MT7 toxin. Views from the side of the TM bundle (Panel A), and from the top of the extracellular space (Panel B). F187^5.41^, F193^5.47^, F281^6.44^, M292^6.55^, F308^7.35^ in green. D106^3.32^ and the double S188^5.42^/S192^5.46^ in orange. F86^2.64^, F288^6.51^ and F312^7.39^ in red. Panel C :3D structure of the three-finger fold MT7 toxin (2vlw) with the four conserved disulfide bridges in red.

We constructed a homology model of the α_1A_-AR based on the β_2_-AR structure [28]. Green, orange and red denote residues with no, moderate or large influence on ρ-Da1a affinity (Fig. 9, panel A and B). Residue D106^3.32^ and the S188^5.42^/S192^5.46^ positions are about 16 Å from the surface of the receptor on one side of the orthosteric site, whereas positions F86^2.64^, F288^6.51^ and F312^7.39^ that all interact strongly with ρ-Da1a are located on the opposite side and distributed from the surface down to a depth of 12 Å. As seen for toxin binding, mutation of F86^2.64^ had a substantial effect on HEAT affinity, whereas mutation of F288^6.51^ had no effect, and mutation of F312^7.39^ to alanine caused a slight increase in the affinity of HEAT. Although we have yet to define additional residues that contribute to HEAT binding, these findings support the idea that ρ-Da1a and the radioligands have overlapping but distinct binding modes.

A three-finger fold toxin can be represented by a 35 Å isosceles triangle of around 10 Å thickness (Fig. 9C). It is therefore much larger than classical small orthosteric ligands but nevertheless, ρ-Da1a is able to interact with positions within the orthosteric cavity of the α_1A_-AR. This is not the case for MT7, as the experimentally-based model of the MT7-M1 muscarinic receptor complex indicates an extracellular location of the toxin involving mainly the extracellular loop e2, in agreement with its allosteric properties [13]. The structural organization of the external part of GPCRs plays an important role in the access to the orthosteric site by agonists. Some receptors, like rhodopsin [53] or the S1P_1_ receptor [54] have their ligand-binding cavity substantially enclosed, compared to the chemokine CXCR4 receptor, for example, in which the extracellular loop conformation renders the binding cavity particularly open, facilitating the binding of large peptides [55]. The top view of the α_1A_-AR model (Fig. 9B) shows a relatively open receptor with external loops on the sides of the receptor, very similar to that proposed for the α_1B_-AR [18]. By comparison, M1 receptor modeling and mutational analysis indicate that extracellular loop 2 is important in binding of both orthosteric and allosteric ligands. The loop shows conformational flexibility but adopts closed conformations that affect access even of small molecule ligands [56]. Residues E170, R171, L174 and Y179 located in the e2 loop of the human M1 receptor collectively interact with MT7, with additional contributions from W91 in the e1 loop and W400 at the top of TM7 [13]. The more open conformation of the α_1A_-AR is certainly consistent with the capacity of ρ-Da1a to interact with residues inside the orthosteric pocket, however the large size and sub-nanomolar affinity of the toxin also suggest additional points of interaction with the receptor. A very recent publication describes how ρ-TIA, a conotoxin of 19 residues which acts as a negative allosteric modulator, interacts with the α_1B_-AR [18]. This small reticulated peptide binds primarily with the extracellular loop e3 of the α_1B_-AR and with the upper part of TM6 and TM7. ρ-TIA affinity is increased by the mutation F310^6.51^A in TM6, whereas our homologous mutation in the α_1A_-AR (F288^6.51^A) decreases ρ-Da1a affinity 18-fold. In TM7, ρ-TIA is sensitive to mutation at position F330^7.35^ of the α_1B_-AR (corresponding to F308^7.35^ in α_1A_-AR, not implicated in ρ-Da1a affinity) but not at position F334^7.39^ (corresponding to F312^7.39^ in α_1A_-AR). Mutation of the α_1A_-AR at residue F312^7.39^, which is one helical turn further from the extracellular face of the α_1A_-AR than F308^7.35^, produces a 93-fold reduction in ρ-Da1a affinity, highlighting the difference in binding mode of ρ-Da1a and the α_1A_-AR compared to ρ-TIA and the α_1B_-AR. Hence of the three animal toxins for which the mode of action has been described, the two negative allosteric modulators (MT7 and ρ-TIA) interact mostly with the external part of their receptor targets while ρ-Da1a interacts with the orthosteric binding site.

We found one discrepancy in our study, namely that ρ-Da1a shows incomplete competition with radioligands in equilibrium binding studies, a property normally characteristic of allosteric modulators. Our combined data, indicate that ρ-Da1a binds at least in part within the orthosteric pocket of the α_1A_-AR, however the large size of the toxin and/or its slow dissociation rate appear to cause altered pharmacology. In yeast membranes expressing the α_1A_-AR, both prazosin and ρ-Da1a cause complete displacement of ^125^I-ρ-Da1a, whereas like in CHO-K1 and COS-7 cells, ρ-Da1a displaces only 85% of ^3^H-prazosin binding [9]. Several three-finger snake toxins display similar incomplete competition for radioligand binding to GPCRs, however in the case of MT7 binding to the M1 muscarinic receptor, this residual binding is readily explained by an allosteric mode of interaction [11], [14], [16]. ρ-Da1b and MTα are also unable to fully inhibit ^3^H-rauwolscine binding to α_2_-ARs despite showing no effect on the ^3^H-rauwolscine dissociation rate, but their modes of action have still not been fully established [10], [57]. A third interesting case is that of ρ-TIA, which has an allosteric mode of action at α_1B_-ARs but has been described as a competitive antagonist of the α_1A_-AR in functional assays [19]. Despite this, ρ-TIA produces only 80% inhibition of ^125^I-HEAT binding in membranes from HEK-293 cells transfected with the α_1A_-AR. We initially thought that all α_1A_-ARs present in membrane preparations may be accessible to small molecule radioligands, but that a sub-population of the receptors might exist in conformations that are inaccessible to the larger toxin. This could reflect steric hindrance or a mixed allosteric/orthosteric mode of action of ρ-Da1a, however if this were the case, and the two populations of receptors were in equilibrium, the residual binding should change over time. We found that this was not the case, as the residual binding showed no time dependence over 2–24 hours. Thus the two receptor pools are not interchangeable, suggesting possible separation between distinct membrane compartments. This question remains to be resolved for ρ-Da1a but also for other toxins that display atypical pharmacological properties.

Key questions arising from our work are to determine which residues of ρ-Da1a bind within the α_1A_-AR orthosteric site, and whether additional regions bind to α_1A_-AR extracellular loops as seen for MT7 and ρ-TIA. Identification of additional receptor binding sites will be of interest because any such extra-orthosteric interaction may contribute to the α_1A_-AR selectivity of ρ-Da1a, as well as the insurmountable antagonism observed here in cell-based assays, on isolated rat [9] or human muscle and in *in vivo* experiments [44]. These questions will be addressed by characterization of mutated ρ-Da1a, by determining the crystal structure of the toxin, and by subsequent docking studies (for example [13]).

In conclusion, our findings demonstrate competitive behavior of the ρ-Da1a toxin at the α_1A_-AR and highlight the crucial role of residues located in the α_1A_-AR orthosteric site for the toxin interaction. Thus, despite the fact that ρ-Da1a and MT7 belong to the same three-finger fold structural family of toxins, and interact with homologous biogenic amine receptors, the mode of interaction with their respective targets is distinct. Evolution of snake toxins has thus not only generated a wide range of pharmacological activities from a unique peptide scaffold, but also various strategies to interact with similar molecular targets.

## Supporting Information

File S1 **Recombinant expression of ρ-Da1a.**
(DOCX)
