# Supplementary material for: Orthosteric Binding of ρ-Da1a, a Natural Peptide of Snake Venom Interacting Selectively with the α1A-Adrenoceptor
Source: PLoS One. 2013 Jul 25;8(7):e68841. doi: 10.1371/journal.pone.0068841 (PMC3723878; doi:10.1371/journal.pone.0068841)
Supplement: File S1 — Recombinant expression of ρ-Da1a. (DOCX) [file pone.0068841.s001.docx]

**File S1 : Recombinant expression of GLY-ρ-Da1a.**

**EXPERIMENTAL PROCEDURES**

*Recombinant expression of ρ-Da1a-* ρ-Da1a was expressed in *E. coli* in a fusion form with the protein ZZ, useful for the expression of the three-finger fold toxins, as previously described (Drevet et al. 1997). The synthetic gene corresponding to the ENLYFQG-ρDa1a protein flanked by the attB1 and attB2 sequences was incorporated by recombination into the pENTRE plasmid according to manufacturer’s protocol (Invitrogene, Cergy Pontoise, France). This was then recombined into the pEXP plasmid to fuse the toxin with the ZZ protein. A Tev cleavage sequence (NLYFQG) was introduced at the N-terminal position of ρ-Da1a to release the final Gly-ρ-Da1a. The gene expression was under the control of the Lac promotor. *E. coli* BL21 start strain, transformed by the plasmid, was grown on LB media, at 37°C until reaching an OD_600nm_ around 0.6. Expression of the fused toxin was induced by Isopropyl β-D-1-thiogalactopyranoside (1mM) for three hours at 37°C. Cells were then centrifuged (4500 g, 20 min.), broken on a Hilton press and centrifuged again (15000 g, 30 min.). Inclusion bodies were solubilized in guanidinium chloride (GndCl) 4 M, Tris 100 mM pH 8 and tris(2-carboxyethyl)phosphine (TCEP) 10 mM, at room temperature for 2 hours. Insoluble particles were removed by centrifugation (18000 g, 60 min.). The supernatant was then diluted 10 times in the Tev buffer (Tris 50 mM pH 8, NaCl 150 mM) supplemented by TCEP 10 mM. Under gentle agitation, 10 mg of Tev per 100 mg of total protein was added for 4 hours at 4°C. The cleaved solution was loaded onto 20 ml of a nickel resin pre-equilibrated with Tris 50 mM pH 8. Resin was washed with Tris 50 mM pH 8 and Gly-ρ-Da1a was eluted with imidazole 50 mM. The fused protein as well as the Tev was eluted with 300 mM imidazole. Protein separation was carried out on SDS-PAGE under denaturating conditions. Gly-ρ-Da1a was diluted 5 times in the folding buffer (Tris 50 mM pH 8, glycerol 25 %, oxidized and reduced gluthation 1 mM) for 36 to 40 h at 4 °C, as previously described (Quinton et al. 2010). The folding solution, after being acidified with trifluoro acid (TFA) was purified by gradient of acetonitrile (ACN) under 0.1 % TFA on a reverse phase column using a waters P600 system (St Quentin en Yvelines, France). The first purification was done over 20 min on a preparative reverse phase column (C18 Vydac, 25 cm*2 cm, 25 µm) at 10 ml/min under a gradient of 20 to 30 % ACN. Folded Gly-ρ-Da1a elutes at around 25 % of ACN, just before part of the contaminant ZZ fusion protein. If necessary, a second run on a semi-preparative column (C5 discover 25 cm * 1 cm, 10 µm) was performed at 4.5 ml/min over 50 min under a gradient of 15 to 40% ACN. Between 4 and 7 mg of pure Gly-ρ-Da1a per liter of culture were obtained. Mass analyses were performed on a Quattro micro (Micromass, Altrincham, UK) by Atheris laboratories (Geneva, Switzerland).

Gly-ρ-Da1a quantifications were obtained by amino acid composition analysis performed under standard conditions. Samples were vaccum dried and sealed in glass tubes using the PicoTag system (Waters Associates, Milford, MA). Samples were then hydrolyzed over 17h at 110°C under vapor phase of 6N HCl with a crystal of phenol. The hydrolyzed sample was recovered with 100 µl of MilliQ water. 90 µl of the HCl hydrolysate (containing a minimum of 200 pmol of each amino acid) were analyzed and quantified via ninhydrin derivatization on an aminoTac JLC-500/V amino acids analyzer (JEOL, Japan). A calibration with an amino acid standard was performed at the beginning of each analyses cycle.

**RESULTS**

*Recombinant production of ρ-Da1a*- We developed a recombinant expression system to produce ρ-Da1a. As we could not produce any toxin coupled with a histidine tag (results not shown), we chose to fuse it with the ZZ protein, as already described for other three-finger fold toxins (Drevet et al. 1997). In addition to the ZZ fusion protein, a histidine tag and a cleavage site specific to the protease Tev were added to obtain the final protein MSYYHHHHHH-ZZ-Tev-ρDa1a (MW = 27487 Da). Whatever the OD of induction (between 0.5 and 1), the induction time (between 3 and 20 hours) or the temperature of induction (between 20 and 37 °C), the MSYYHHHHHH-ZZ-Tev-ρDa1a protein was always produced in inclusion bodies, with a yield between 30 and 50 mg per liter of culture. We induced protein expression at an OD_600_ of around 0.6, by adding 1 mM IPTG for 3 h at 37°C. Inclusion bodies were solubilized in GdnCl buffer in the presence of a reducing agent (TCEP 10 mM) to limit precipitation during dilution. The fused protein migrated with an apparent molecular weight of 30 kDa (SI Fig. 1A, column 2). The dilution phase was necessary to maintain an efficient Tev enzymatic activity, which decreases under GndCl concentrations higher than 500 mM. 10 mg of Tev for 100 mg of total proteins was used to reach a 70 % yield of cleavage (SI Fig. 1A, column 3). Gly-ρ-Da1a was purified from fusion protein and Tev using a nickel affinity column. Gly-ρ-Da1a was eluted with 50 mM of imidazole (SI Fig. 1A, column 4 and 5. Fig. 1B) and the tagged proteins were eluted with 300 mM of imidazole (SI Fig. 1A, column 6. Fig.1B). The linear peptide was then diluted in folding buffer for 36 to 40 h before being loaded onto a preparative column. Elution of Gly-ρ-Da1a occurred at around 26 % of acetonitrile (SI Fig. 1C). The rising part of the left peak is composed of pure Gly-ρ-Da1a while its right part is contaminated with ZZ fusion protein (SI Fig. 1C). This right part was further purified on a semi-preparative column (not shown). Gel SDS-PAGE and mass spectrometry analysis were performed to check that the purity was higher than 95 %. Gly-ρ-Da1 displays the same affinity as the synthetic ρ-Da1a, thus excluding a functional role of the additional N-terminal glycine (Fig. 1A).

FIGURE 1 **Recombinant production and purification of Gly-ρ-Da1a**. A: SDS-PAGE of the different fractions obtained during the production and purification of Gly-ρ-Da1a. Column 1 and 7: molecular weight markers. Column 2 and 3: crude inclusion bodies before and after Tev cleavage, respectively. Column 4 and 5: 50 mM imidazole elution. Column 6: 300 mM imidazole elution. B: Nickel affinity purification of free and tagged Gly-ρ-Da1a protein. Gly-ρ-Da1a was in peak marked by an arrow. C: Purification of the oxidized Gly-ρ-Da1a by reverse phase chromatography. The refolded ρ-Da1a was eluted at 26% ACN (peak marked by an arrow).

Drevet, P., Lemaire, C., Gasparini, S., Zinn-Justin, S., Lajeunesse, E., Ducancel, F., Pinkasfeld, S., Courcon, M., Tremeau, O., Boulain, J. C. ,Menez, A., 1997. High-level production and isotope labeling of snake neurotoxins, disulfide-rich proteins. Protein Expr Purif 10, 293-300.

Quinton, L., Girard, E., Maiga, A., Rekik, M., Lluel, P., Masuyer, G., Larregola, M., Marquer, C., Ciolek, J., Magnin, T., Wagner, R., Molgo, J., Thai, R., Fruchart-Gaillard, C., Mourier, G., Chamot-Rooke, J., Menez, A., Palea, S., Servent, D. ,Gilles, N., 2010. Isolation and pharmacological characterization of AdTx1, a natural peptide displaying specific insurmountable antagonism of the alpha(1A)-adrenoceptor. Br J Pharmacol 159, 316-325.
